# *In vivo* dual targeting of the oncogenic *Ether-à-go-go*-1 potassium channel by calcitriol and astemizole results in enhanced antineoplastic effects in breast tumors

**DOI:** 10.1186/1471-2407-14-745

**Published:** 2014-10-03

**Authors:** Janice García-Quiroz, Rocío García-Becerra, Nancy Santos-Martínez, David Barrera, David Ordaz-Rosado, Euclides Avila, Ali Halhali, Octavio Villanueva, Maŕa J Ibarra-Sánchez, José Esparza-López, Armando Gamboa-Domínguez, Javier Camacho, Fernando Larrea, Lorenza Díaz

**Affiliations:** Departamento de Biología de la Reproducción, Instituto Nacional de Ciencias Médicas y Nutrición Salvador Zubirán, Vasco de Quiroga No. 15, Tlalpan, México, DF 14000 México; Departamento de Farmacología, Centro de Investigación y de Estudios Avanzados del I.P.N., Av. Instituto Politécnico Nacional No. 2508, Gustavo A. Madero, México, DF 07360 México; Departamento de Investigación Experimental y Bioterio, Instituto Nacional de Ciencias Médicas y Nutrición Salvador Zubirán, México, DF México; Departamento de Bioquímica, Instituto Nacional de Ciencias Médicas y Nutrición Salvador Zubirán, México, DF México; Departamento de Patología, Instituto Nacional de Ciencias Médicas y Nutrición Salvador Zubirán, México, DF México

**Keywords:** Vitamin D, Breast cancer, Vitamin D receptor, Targeted therapy, Ki-67, EAG1

## Abstract

**Background:**

The oncogenic *ether-à-go-go-1* potassium channel (EAG1) activity and expression are necessary for cell cycle progression and tumorigenesis. The active vitamin D metabolite, calcitriol, and astemizole, a promising antineoplastic drug, target EAG1 by inhibiting its expression and blocking ion currents, respectively. We have previously shown a synergistic antiproliferative effect of calcitriol and astemizole in breast cancer cells *in vitro*, but the effect of this dual therapy *in vivo* has not been studied.

**Methods:**

In the present study, we explored the combined antineoplastic effect of both drugs *in vivo* using mice xenografted with the human breast cancer cell line T-47D and a primary breast cancer-derived cell culture (MBCDF). Tumor-bearing athymic female mice were treated with oral astemizole (50 mg/kg/day) and/or intraperitoneal injections of calcitriol (0.03 μg/g body weight twice a week) during 3 weeks. Tumor sizes were measured thrice weekly. For mechanistic insights, we studied EAG1 expression by qPCR and Western blot. The expression of Ki-67 and the relative tumor volume were used as indicators of therapeutic efficacy.

**Results:**

Compared to untreated controls, astemizole and calcitriol significantly reduced, while the coadministration of both drugs further suppressed, tumor growth (*P* < 0.05). In addition, the combined therapy significantly downregulated tumoral EAG1 and Ki-67 expression.

**Conclusions:**

The concomitant administration of calcitriol and astemizole inhibited tumor growth more efficiently than each drug alone, which may be explained by the blocking of EAG1. These results provide the bases for further studies aimed at testing EAG1-dual targeting in breast cancer tumors expressing both EAG1 and the vitamin D receptor.

## Background

Breast cancer is the most frequently diagnosed malignant neoplasia and the leading cause of cancer death among women worldwide [1]. One out of eight women will develop breast cancer during their lifetime. The standard medical treatment for breast cancer besides surgery and radiotherapy include cytotoxic chemotherapy, which targets rapidly dividing cells. However, this clinical approach is highly toxic, affects normal cells and causes a wide array of side effects. In the last decades, substantial changes in cancer therapy have been made. Among them, new anticancer drugs designed to recognize specific features in cancer cells are being produced. These drugs, created on the basis of their targeted mechanism of action, are expected to be more efficient with less toxicity. Approximately 80% of all breast cancers are susceptible for hormonal or antibody-based targeted therapy, based on the presence and/or abundance of the estrogen receptor alpha (ERα), progesterone receptor (PR) and/or the human epidermal growth factor receptor 2 (HER2). It is well known that absence of ERα, PR and HER2 precludes targeted therapies to these cell markers and often results in poorer outcomes [2]. Therefore, identification of new molecular targets expressed in breast tumors is needed. The *ether à-go-go-1* potassium channel (EAG1) became an oncological target soon after the discovery of its involvement in cell proliferation and apoptosis [3–6]. EAG1 promotes oncogenesis and tumor progression, and its pharmacological inhibition reduces tumor development [4, 6, 7]. Moreover, EAG1 is upregulated by cancer-associated factors such as estrogens and the human papilloma virus [8]. Interestingly, a substantial proportion of breast tumors including ERα-negative and triple-negative breast cancers express EAG1 [5, 9]. In this regard, the progression of breast cancer cells through the early G1 phase has been shown to be dependent on the activation of EAG1 channels [10–12]. Previously, our laboratory showed that EAG1 expression and the rate of cell proliferation are inhibited in breast and cervical cancer cells by calcitriol, the active vitamin D metabolite [9, 13]. Calcitriol is an important endogenous as well as exogenous anticancer hormone. The antiproliferative effects of calcitriol have been extensively demonstrated in many cancerous cell types, most of them involving the ligand-activated vitamin D receptor (VDR) [14, 15]. Since the induction of cell cycle arrest and apoptosis by calcitriol depends on the expression of the VDR, this protein represents a good therapeutic target in treating cancer [16].

Previous *in vitro* studies by our group have shown that astemizole, a non-selective EAG1 blocker, synergized with calcitriol to inhibit breast cancer cell proliferation by modifying EAG1 gene expression and possibly its activity as well [17]. In addition, these studies also showed that astemizole upregulates VDR expression and downregulates the calcitriol-degrading enzyme CYP24A1; thus, increasing calcitriol bioactivity while decreasing its degradation. Taken together these observations and the fact that the VDR and EAG1 are expressed in 90% and 85%; respectively, of breast cancer tumors [18–21], we hypothesized that a combined treatment targeting these two proteins *in vivo* could result in an improved therapeutic benefit for breast cancer management, including those tumors not treatable by hormonal therapy. In the present study we investigated the effects of calcitriol alone or in combination with astemizole on tumor growth in an *in vivo* preclinical model using athymic mice xenografted with two different human breast cancer cell lines: T-47D (ERα, VDR and EAG1 positive) and a ductal infiltrating carcinoma breast cancer-derived primary cell culture (MBCDF, ERα negative, VDR and EAG1 positive) [22]. These two cell lines were selected because they represent different types of breast tumors based on the expression of the ERα. In addition, both express the selected therapeutic targets and both were tumorigenic. Herein, we show for the first time that the concomitant *in vivo* administration of calcitriol and astemizole inhibited tumor growth more efficiently than each drug alone.

## Methods

### Reagents

Calcitriol (1,25-dihydroxycholecalciferol) was kindly donated from Hoffmann-La Roche Ltd (Basel, Switzerland). Astemizole was acquired as a pediatric suspension from the local pharmacy (Astesen® Senosiain Laboratories).

### Breast cancer cell culture

The MBCDF primary breast cancer cell culture was generated from a biopsy obtained from a radical mastectomy performed on a patient with an infiltrating ductal carcinoma stage IV. The protocol was approved by the Human Research Ethics Committee from the Instituto Nacional de Ciencias Médicas y Nutrición Salvador Zubirán (INCMNSZ) in Mexico City (Ref 1549, BQO-008-06/9-1) [22] and written informed consent was obtained from the patient. Cells were maintained in humidified atmosphere with 5% CO_2_ at 37°C in RPMI-1640 medium supplemented with 100 units/mL penicillin plus 100 μg/mL streptomycin and 5% heat-inactivated fetal bovine serum. The established human breast cancer cell line T-47D was also used in this study (ATCC, Manassas, VA) and was maintained following indications from the supplier.

### Immunocytochemistry

Cultured cells were grown on glass coverslips and fixated in ethanol 96%. Antigen retrieval was done by autoclaving in EDTA (0.1 M, pH 9.0). Slides were blocked with immunodetector peroxidase blocker (Bio SB, Santa Bárbara CA, USA). For EAG1, additional blocking was performed using background Sniper (Biocare Medical, CA, USA). The following primary antibodies were incubated for 2 hours: Anti- ERα (1:250, Bio SB), anti-VDR (1:100, Santa Cruz Biotechnology Inc, CA, USA) and anti-EAG1 (1:300, Novus Biologicals CO, USA). After washing, the slides were sequentially incubated with immuno-Detector Biotin-Link and immuno-Detector HRP label (Bio SB) during 10 minutes each. Staining was completed with diaminobenzidine (DAB) and slides were counterstained with hematoxylin.

### Induction of tumors in athymic mice

Studies involving mice were performed according to the rules and regulations of the Official Mexican Norm 062-ZOO-1999. The study was approved by the Institutional Committee for the care and use of laboratory animals (protocol number BRE-31-10-13-1, CINVA 31) of the INCMNSZ, where mice were housed in the animal facility. Athymic female BALB/c homozygous, inbred Crl:NU(NCr)-Foxn1nu nude mice (~6 weeks of age) were kept in ventilated cages (34 air changes hourly) with bedding of aspen wood-shavings, controlled temperature, humidity and 12:12 light:dark periods. Sterilized water and feed (standard PMI 5053 feed) were given *ad libitum*. Appropriate animal observations were made in order to minimize/alleviate any potential pain, distress, or discomfort by choosing the earliest endpoint compatible with the scientific objectives of this work. Tumors were induced by subcutaneous injection of MBCDF or T-47D cells (2.0 x 10^6^) in 0.1 mL of sterile saline solution into the upper part of the posterior limb of each mouse.

### Therapeutic protocol

When the tumors reached a palpable mass (~3 mm), mice were randomly divided in 4 groups and received either: vehicle (i.p. ethanol, 1.8 μL/100 μL of sterile saline solution), calcitriol (i.p. 0.03 μg/g of body weight every Tuesday and Thursday), astemizole (p.o. 50 mg/kg/day), or calcitriol + astemizole during 3 weeks. At least 15 mice were included in each group. The dosage of the drugs and the intermittent calcitriol administration regimen were based on published observations [23, 24]. The suspension of astemizole was diluted in the drinking water of mice. Each mouse is estimated to drink 5–7 mL of water per day, which was taken into consideration to achieve as close as possible the dose of astemizole [25]. This supplemented water was changed every day. Weight loss was used as a parameter for toxicity; thus, mice were weighed three times per week to determine any toxic effect of the drugs. Tumor sizes were also measured thrice weekly throughout the experiment. Tumors were measured with a caliper always by the same person. Tumor volume was calculated using the standard formula (length x width^2^)/2, where length is the largest dimension and width the smallest dimension perpendicular to the length. Fold increase from initial volume was calculated for each single tumor by dividing the tumor volume on day 21 by that on day 0 (which corresponded to the tumor volume in the first day of treatment, and was set to one).

### Imunohistochemistry

Tumoral tissue was collected upon termination of the study and immediately fixed in 10% aqueous formaldehyde followed by routine paraffin embedding procedures. Two-micrometer sections were cut, dewaxed in xylene and re-hydrated with descending concentrations of ethanol. Antigen retrieval was done by autoclaving in ImmunoDNA Retriever Citrate (Bio SB). Slides were blocked with PolyDetector Peroxidase Blocker (Bio SB) and then incubated in the presence of a monoclonal anti-Ki-67 antibody (1:100, Bio SB). Next, the slides were incubated with Immuno-Detector HRP label (Bio SB) and staining was completed with DAB. After identifying areas with the most intensive staining, counting of Ki-67- positive cells was done in three different fields per tumor slide in pictures taken with the 20 X objective. Herein, to reduce the subjectivity, three independent observers participated in the counting procedure of Ki-67 positive cells. Afterwards, average number of stained cells was calculated for each group.

### Real Time PCR (qPCR)

Total RNA was extracted by homogenizing the tissue in the presence of Trizol reagent (Life Technologies, Carlbad, USA). Two μg of total RNA were reverse-transcribed and resulting cDNAs were used for the qPCR. The reverse transcription system and the TaqMan Master reagents were from Roche (Roche Applied Science, IN, USA). Amplifications were carried out in the LightCycler® 2.0 from Roche, according to the following protocol: activation of Taq DNA polymerase and DNA denaturation at 95°C for 10 min, followed by 45 amplification cycles consisting of 10 s at 95°C, 30 s at 60°C, and 1 s at 72°C. Gene expression of the housekeeping genes glyceraldehyde-3-phosphate dehydrogenase (GAPDH) or β-actin was used as internal control for T-47D and MBCDF, respectively. Primers sequences were as follows: human EAG1 (hEAG1) [GenBank:AF078741.1]: cct gga ggt gat cca aga tg/cca aac acg tct cct ttt cc; GAPDH [GenBank:AF261085.1]: agc cac atc gct gag aca c/gcc caa tac gac caa atc c and β-actin [GenBank:NM_001101.3]: cca aac cgc gag aag atg a/cca gag gcg tac agg gat ag. Corresponding probe numbers from the universal probe library (Roche) were: 49, 60, and 64 for hEAG1, GAPDH and β-actin respectively. A sample of brain was also processed to test mouse EAG1 expression in this tissue (mEAG1) [GenBank:NM_001038607.1]. For this we used: acg ctt ttg aga acg tgg at/ccg cac aac ttt cag aga act; and the housekeeping gene *mus musculus* ribosomal protein L32 (mL32) [GenBank:NM_172086.2]: gct gcc atc tgt ttt acg g / tga ctg gtg cct gat gaa ct with corresponding probes No. 66 and 46, respectively. In all cases the expression of the gene of interest was normalized against the housekeeping gene and control values were arbitrarily set to one.

### Determination of serum total calcium

Blood samples from three mice in each experimental group were obtained by cardiac puncture under anesthesia, causing exsanguination until the animal death. Total serum calcium concentration was determined by indirect potentiometry using a calcium selective electrode in conjunction with a sodium reference electrode (Synchrom Clinical System CX5 PRO, Beckman Coulter Inc., Fullerton, CA, USA).

### Western blot

A piece of the excised tumors was homogenized with RIPA buffer (9.1 mM dibasic sodium phosphate, 1.7 mM monobasic sodium phosphate, 150 mM NaCl, 1% Nonidet P-40, 0.1% SDS, pH 7.4) in the presence of a protease inhibitor cocktail (Roche) using a Polytron homogenizer (BioSpec Products, Inc.). Thirty micrograms of total tissue lysates were separated on 10% SDS-PAGE, transferred to Immobilon-P PVDF membranes (Millipore, Billerica, MA) and blocked with 5% non-fat milk in PBS-Tween. Membranes were incubated with respective antibodies at appropriate dilutions: anti-EAG1 (Novus Biologicals CO, 1:300) and anti-GAPDH (Millipore, Temecula, CA, 1:10,000). For visualization, membranes were incubated with respective horseradish peroxidase-conjugated secondary antibodies (1:10,000) and were processed with the ECL + Plus Western blotting detection system (GE-Healthcare, UK). Densitometric analysis of resulting bands was performed by using ImageJ software (NIH, USA).

### Statistical analysis

Statistical differences for dose–response assays were determined by One-Way ANOVA followed by Holm-Sidak for pair-wise comparisons (SigmaStat, Jandel Scientific). Differences were considered statistically significant at *P* < 0.05.

## Results

### Cell characterization

**Figure 1:**
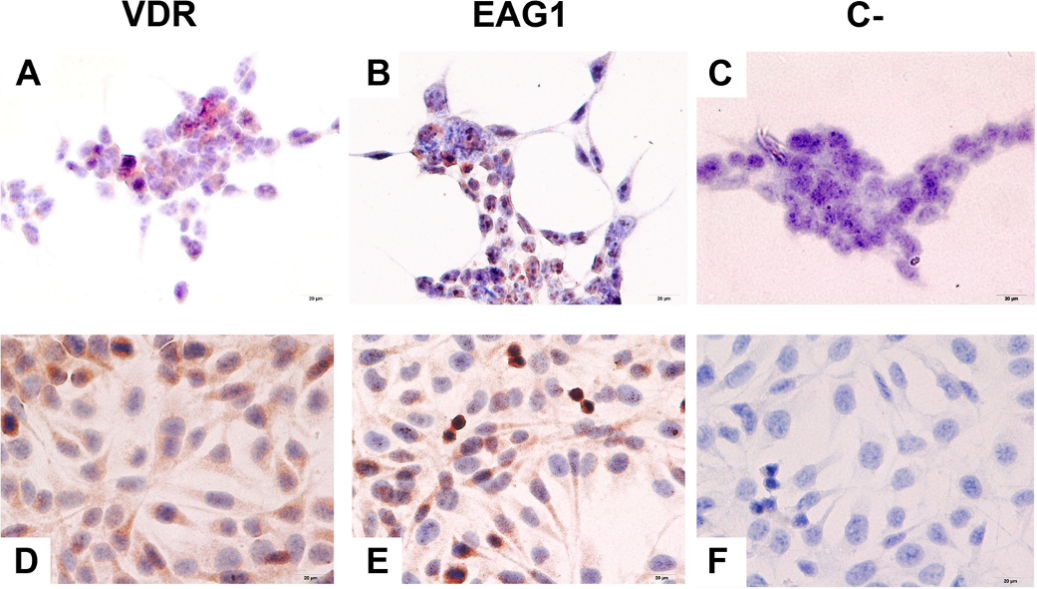
**T-47D and MBCDF cell lines express VDR and EAG1.** The cell lines used in this study T-47D **(A-C)** and MBCDF **(D-F)** express both biomarkers for the targeted therapy. Cells were incubated in the presence of anti-VDR **(A, D)**, anti-EAG1 **(B, E)** or without first antibody **(C, F)** as negative control (C**-**) and further processed as described under Materials and Methods. Positive immunoreactivity is shown in brown staining. Representative pictures are shown (40 X).

The cell lines MBCDF and T-47D were representative of different breast cancer subtypes, based on the differential expression of ERα. Whereas MBCDF and T-47D were negative and positive for ERα, respectively, (data not shown), both cell lines expressed VDR and EAG1 (Figure 1, brown staining).

### Analysis of the tumor volume and Ki-67 expression

**Figure 2:**
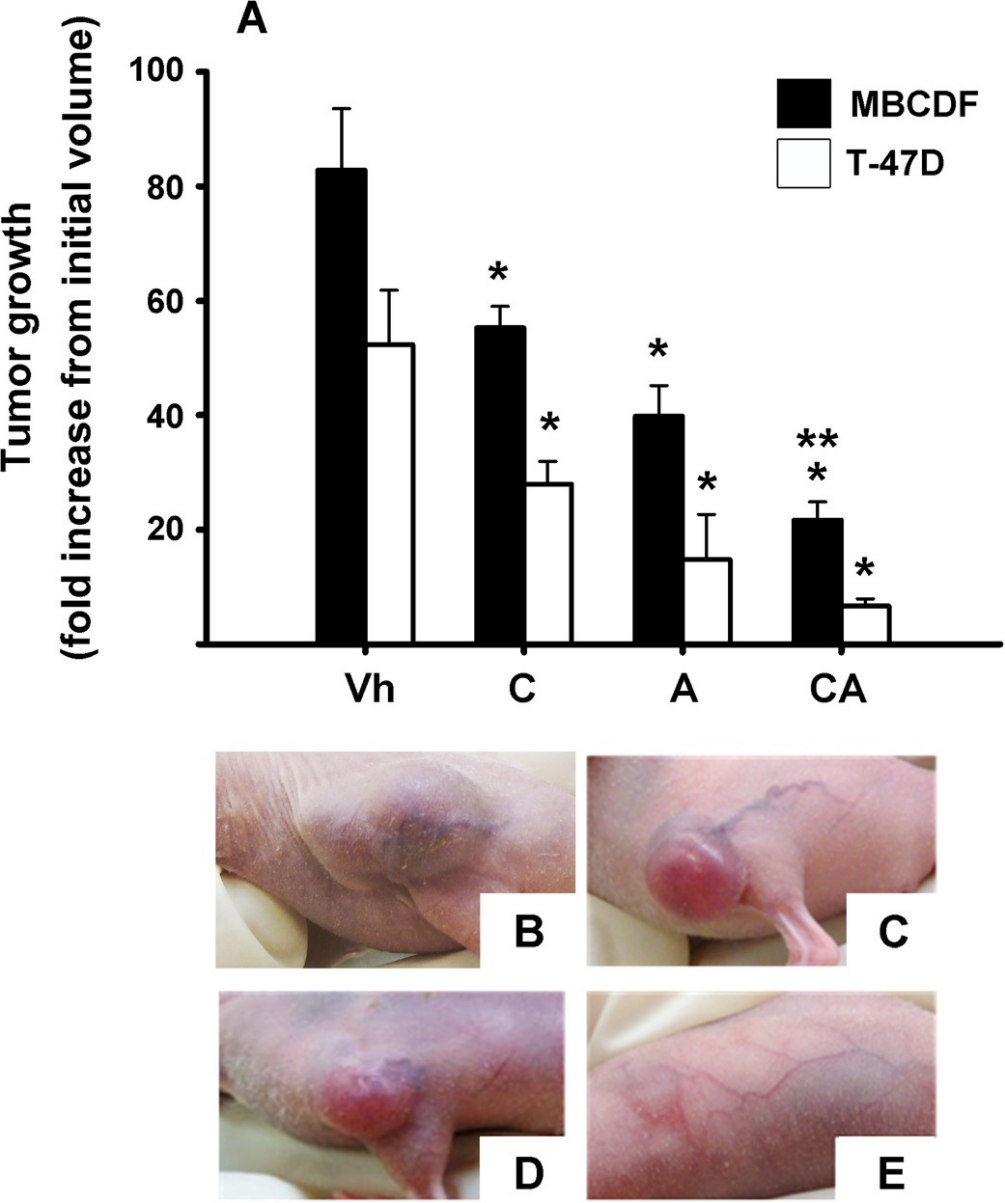
**The combined therapy slows the growth of tumors more efficiently than each drug individually.** MBCDF and T-47D cells were xenografted subcutaneously in nude mice. Treatment started after a palpable mass was evident (initial volume) and was administered during 3 weeks. **A)** Fold increase from initial volume was calculated for each tumor by dividing the volume on day 21/initial volume. Vehicle (Vh); calcitriol (C), astemizole (A), combined therapy (CA). Black bars = MBCDF; white bars = T-47D. Mean ± standard error. *P < 0.05 *vs* vehicle in each group, **P < 0.05 *vs* calcitriol alone. n ≥ 15 mice in each group. **B**, **C**, **D** and **E)** Representative pictures depicting tumor final size after treatment with vehicle, calcitriol, astemizole and the combination of both drugs, respectively.

**Figure 3:**
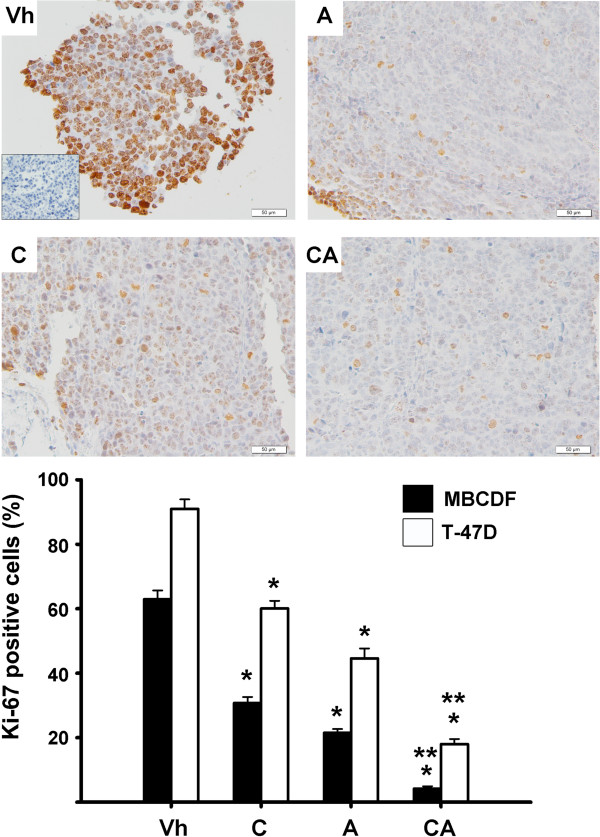
**Ki-67 was expressed by less tumoral cells in mice with combined therapy compared to monotherapy.** Ki-67-positive cells in the tumors were counted in slides from the groups of mice treated with: vehicle (Vh), calcitriol (C), astemizole (A) or their combination (CA). Upper panel: Representative pictures of T-47D tumors from mice treated with the different drugs, showing immunohistochemistry studies of Ki-67 (nuclear, brown staining). Inset shows negative control in the absence of first antibody. All pictures are 20 X. Lower panel: Graphical representation of positive Ki-67 cells in immunohistochemistry slides (%). Black bars = MBCDF; white bars = T-47D. After identifying areas with the most intensive staining, counting of total cells was done in three different fields per tumor slide and the percentage of Ki-67- positive cells was calculated. Results are expressed as the mean ± standard error. * *P* < 0.05 *vs* vehicle in each group**.** **P < 0.05 *vs* each drug alone.

Two groups of nude mice were xenografted subcutaneously either with MBCDF or T-47D tumorigenic breast cancer cells and then randomly assigned to the different treatments. The tumor volume analysis showed that calcitriol and astemizole significantly reduced tumor growth *per se* in both groups of mice (MBCDF and T-47D) compared to controls; however, the co-administration of the two drugs further slowed tumor progression (Figure 2). Accordingly, as shown in Figure 3, the percentage of Ki-67-positive tumor cells was significantly lower in mice treated either with astemizole or calcitriol alone compared to control mice, and it was even lower in tumors from mice treated with both drugs simultaneously.

### The combined treatment with calcitriol and astemizole downregulated EAG1 expression in the tumor tissue

**Figure 4:**
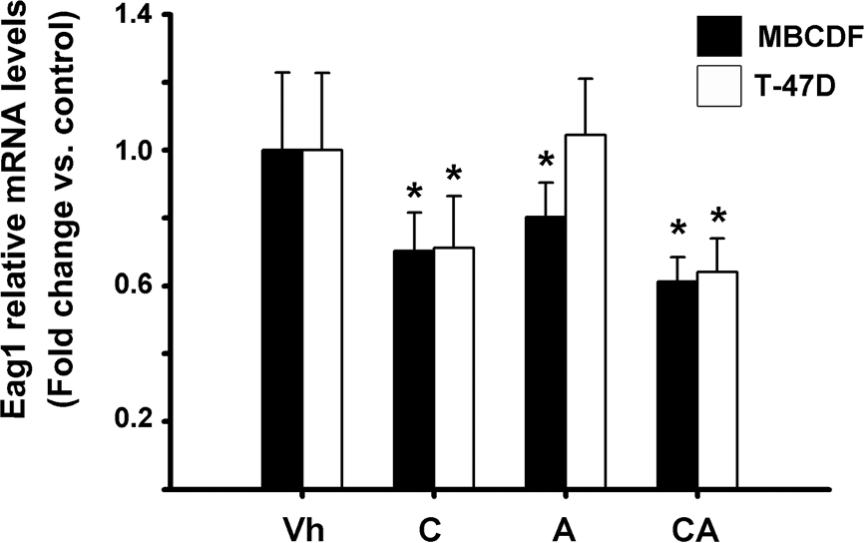
**The combined treatment with calcitriol and astemizole downregulated EAG1 gene expression in the tumoral tissue.** The gene expression of tumoral EAG1 was assessed by RT-qPCR and normalized against the corresponding housekeeping gene. Values for controls were arbitrarily set to one. Black bars = MBCDF**;** white bars = T-47D. *P < 0.05 *vs.* control in each group; n ≥ 15. Results are expressed as the mean ± standard error.

**Figure 5:**
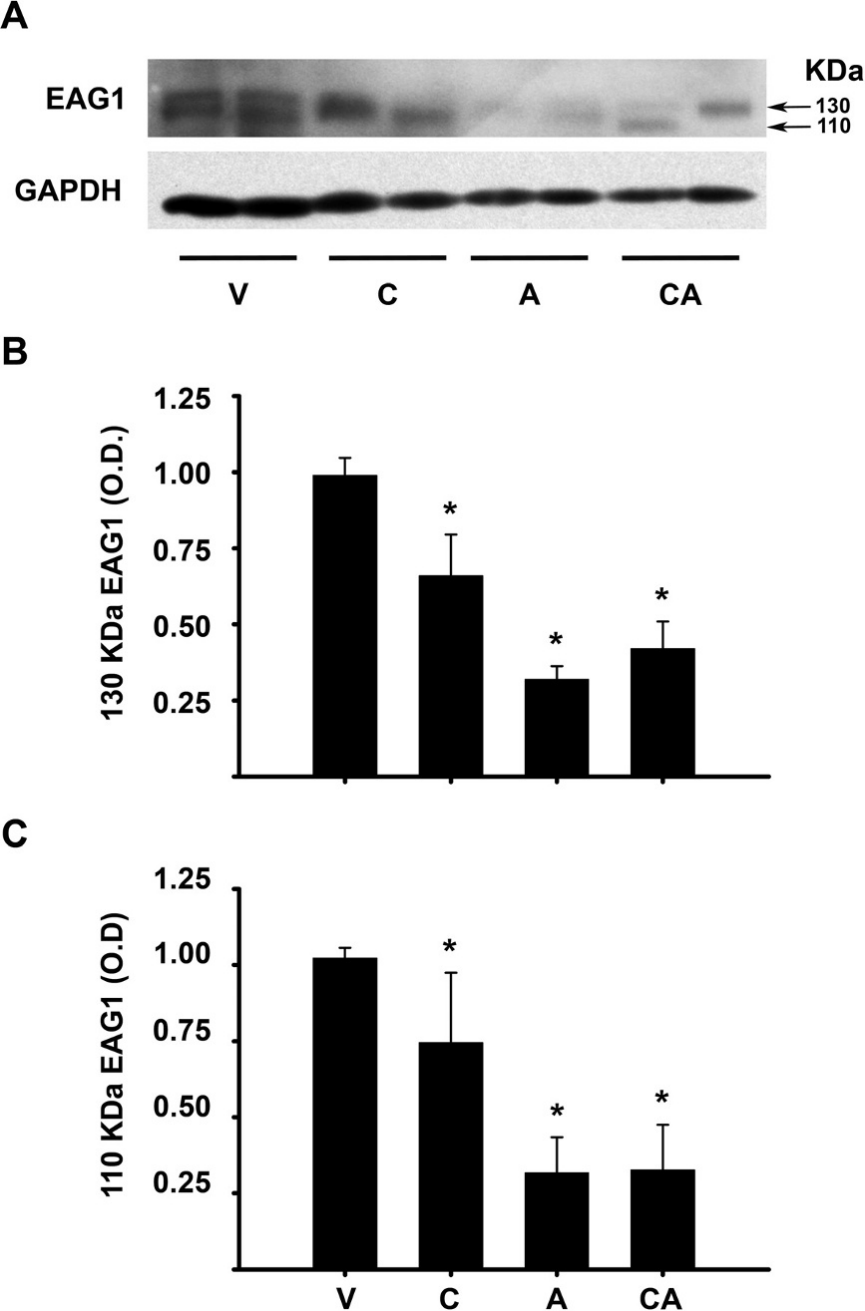
**Less tumor EAG1 protein expression was found in mice treated with the combined treatment. A)** Representative Western blot of EAG1 expression showing two different T-47D tumors per treatment. The mice bearing palpable tumors received vehicle (Vh); calcitriol (C), astemizole (A) or the combination of both drugs (CA) for 3 weeks. Tumors were excised, homogenized and 30 μg of total protein were loaded per lane. Two immunoreactive bands of ~110 kDa and 130 kDa (indicated by arrows) were detected. **B)** and **C)** represent the quantification of the 130 kDa and 110 kDa bands, respectively. The expression of each of the two species of EAG1 was normalized against GAPDH optical density (O.D) and results are shown as the mean ± standard deviation of EAG1 relative O.D of four different tumors from each treatment. *P < 0.05 vs. Vh. Vehicle was given an arbitrary value of 1.

Since previous studies showed that one of the mechanisms involved in calcitriol anticancer effects in breast cancer was its ability to inhibit EAG1 expression [9, 17], we explored whether this process could also be taking place in our *in vivo* model. As shown in Figure 4, calcitriol *per se* significantly downregulated tumor EAG1 gene expression, which was further reduced when calcitriol and astemizole were administrated simultaneously. Next, we examined whether tumor EAG1 protein levels were also affected by the treatments. For this, EAG1 protein levels were evaluated by Western blots of proteins extracted from tumors derived from T-47D cells (Figure 5). As depicted, two distinct bands were detected in the cell homogenates, with an electrophoretic mobility corresponding to ∼ 110 and ∼ 130 kDa, respectively (Figure 5A), which might represent differential glycosylation patterns of asparagines at positions 388 and 406 of EAG1, as described elsewhere [26]. As shown in Figures 5B and C, the treatments reduced significantly EAG1 protein expression when compared to mice treated with vehicle. An unexpected finding was the effect of astemizole by itself on EAG1 mRNA and protein expression (Figures 4 and 5).

Interestingly, neither pharmacological agent alone or combined modified EAG1 gene expression in a tissue that normally expresses this ion channel, such as the brain. The relative expression of EAG1 mRNA in the brains of vehicle, calcitriol, astemizole and both drugs -treated animals was: 0.090 ± 0.024, 0.095 ± 0.023, 0.103 ± 0.024 and 0.095 ± 0.016, respectively; n ≥ 5; *P* = 0.746.

### Effects of calcitriol/astemizole treatment on serum levels of total calcium and body weight

Calcium serum levels and body weight were used as parameters to evaluate specific calcitriol side effects. Mean serum total calcium levels were not affected by the treatments since calcemia was not significantly different among groups (*P* = 0.06). However, the group that received the combined regimen of calcitriol plus astemizole had the highest serum calcium concentration (Table 1). On the other hand, the final body weights were not significantly different among the treated and control groups (Table 1, *P* = 0.09), further indicating the lack of relevant adverse side effects of the drugs used herein at the doses tested, upon these two variables.

**Table 1:** **Total calcium in mice serum and final body weights**

Group	Control	Calcitriol	Astemizole	C + A
**Total calcium (mg/dL)**	9.8 ± 0.2	9.4 ± 0.7	10.0 ± 0.2	10.7 ± 0.6
**Mean final body weight (g)**	22.0 ± 2.0	21.4 ± 2.0	22.6 ± 2.8	21.0 ± 2.6

For calcium: data are expressed as the mean ± S.D., n = 3. The mean final body weight was calculated including both T-47D and MBCDF inoculated mice and are presented as the mean ± S.D., n ≥ 24.

In addition, mice treated with astemizole showed a healthy and soft skin, while the calcitriol-treated mice showed mild dry skin.

## Discussion

For a patient tailored anticancer therapy, the identification of potential targets in tumor tissue is paramount to predict therapeutic efficiency. Indeed, new antineoplastic drugs are expected to be less toxic to the patient and more tissue- and target-specific. Although some molecular subgroups of breast cancer are beneficed from a targeted therapy, the most aggressive tumors still lack molecular targets, representing a clinical challenge. Among the newly recognized therapeutic targets in oncology, EAG1 stands as a promising candidate, considering its involvement in oncogenesis and tumor growth [27]. Other important therapeutic target is the VDR; which is required to mediate calcitriol antineoplastic effects, such as the repression of EAG1 gene expression [9, 13, 28, 29]. Therefore, drugs that target the VDR and EAG1 represent novel approaches to fight against breast cancer. Interestingly, both EAG1 and the VDR are expressed in most breast tumors, independently of their general molecular signature [18–21]. Considering the latter, we designed a combined targeted therapy directed to these biomarkers *in vivo*, based on the rationale of a dual blocking of EAG1 with the purpose to restrain its tumorigenic ability and consequently, tumor progression. For this, we used astemizole and calcitriol to inhibit EAG1 activity and gene expression; respectively, in order to completely obstruct EAG1 functionality. The design of the study was conceived to be tested in a murine model xenografted with two human breast cancer cell lines expressing both EAG1 and the VDR. Tumor growth reduction together with Ki-67 expression as proliferation marker, were used as biological endpoints representing therapeutic benefit. Taking into consideration these endpoints, the results showed that the combined regimen was significantly more efficient to produce antitumor effects than when either of the agents was tested alone. In addition, calcitriol significantly inhibited tumoral EAG1 mRNA and protein expression, an effect that was further increased by the co-administration with astemizole, showing for the first time the *in vivo* inhibition of this oncogenic potassium channel by these drugs in breast tumors.

Interestingly, the expression of EAG1 was also inhibited by astemizole, in a similar manner as observed previously *in vitro* [17]. This could probably be explained by astemizole blocking EAG1 channels located also in the inner nuclear membrane, which has been suggested to affect gene expression [30].

Besides the double inhibition of EAG1 by the combined treatment, additional individual antiproliferative effects of astemizole and calcitriol might also be taking place, such as long-term blocking of histamine H_1_-receptors, induction of apoptosis or the modification of EAG1 activity and/or glycosylation patterns, which deserve to be further investigated.

Since calcitriol bioavailability and activity are potentiated by astemizole [17], its effects on calcium serum levels should be considered and avoided to prevent hypercalcemia, as an undesirable side effect. In this study, only the combined therapy was accompanied by a mild increase in serum calcium levels, which probably resulted from improved calcitriol bioactivity. In addition, no changes in the mean body weights between the experimental and control groups were observed, and the histopathological analysis of the lungs of treated animals by a specialized pathologist did not show any signs of toxicity, suggesting that the doses of calcitriol and astemizole, as used in this study, were well tolerated. These data suggested that the combined dosing regimen herein reported could potentially be tested in patients with breast cancer as an adjuvant therapy, with relative low adverse side effects. Alternatively, dietary vitamin D instead of calcitriol could be used since it is a safe, economical and easily available nutritional agent, that has proven to be equivalent to calcitriol in exerting anticancer effects in a preclinical model of breast cancer [31]. On the other hand, many different compounds may be used to target EAG1; however, for the purposes of this study we chose astemizole, given its well-known *in vitro* and *in vivo* antiproliferative effects on tumor cells through blocking ion currents [11, 17, 23, 32]. In addition, astemizole offers other advantages, such as the fact that it is a low-priced drug currently prescribed for treatment of simple allergic conditions or malaria in some countries and particularly because it does not cross the blood–brain barrier [33]. Regarding this, the observation that neither astemizole nor calcitriol modified the expression of EAG1 in the brains of the treated mice, as they did in breast cancer, was of particular importance since normal brain cells express EAG1. This observation may rule out alterations on the physiological role of EAG1 at the level of the central nervous system.

Overall, our results confirm previous *in vitro* findings [9, 17] and support earlier studies showing EAG1 as a promising target for the tailored treatment of human tumors [21]. As previously suggested, reevaluation of astemizole as an antineoplastic drug is needed [6].

In summary, in this study using an *in vivo* preclinical animal model of breast cancer, the combined administration of calcitriol with astemizole improved significantly their individual therapeutic efficiency in terms of tumor growth inhibition. This effect could be explained by the dual inhibitory effect on EAG1 and increased calcitriol bioactivity. Since both astemizole and calcitriol inhibit EAG1 activity and expression, respectively, patients bearing EAG1 and VDR-positive solid or metastatic tumors may benefit from this EAG1 double blocking strategy.

## Conclusions

The simultaneous administration of calcitriol and astemizole to mice xenografted with human breast cancer cells reduced tumor growth more efficiently than either drug alone. The mechanistic explanation for these results includes the inhibition of EAG1 expression, providing scientific bases to test the combined therapy in future clinical trials. The effect of the combined treatment was effective in both types of tumors (ER negative and ER positive), as seen in MBCDF and T-47D-xenografts, further supporting the potential use of this therapeutic approach in breast tumors that represent a clinical challenge, such as triple negative and those resistant to endocrine therapy. Some of the limitations of this study include the lack of serum calcitriol quantification and the analysis of tumoral VDR and CYP24A1 expression, which deserve to be further investigated.

## Abbreviations

EAG1: Ether-à-go-go-1 potassium channel
VDR: Vitamin D receptor.
